# Assays of CFTR Function In Vitro, Ex Vivo and In Vivo

**DOI:** 10.3390/ijms23031437

**Published:** 2022-01-27

**Authors:** Anabela S. Ramalho, Mieke Boon, Marijke Proesmans, François Vermeulen, Marianne S. Carlon, Kris De Boeck

**Affiliations:** 1 CF Research Lab, Woman and Child Unit, Department of Development and Regeneration, KU Leuven (Catholic University of Leuven), B-3000 Leuven, Belgium; mieke.boon@uzleuven.be (M.B.); marijke.prosmans@uzleuven.be (M.P.); francois.vermeulen@uzleuven.be (F.V.); christiane.deboeck@uzleuven.be (K.D.B.); 2 Department of Pediatrics, Pediatric Pulmonology, University Hospital of Leuven, B-3000 Leuven, Belgium; 3 Molecular Virology and Gene Therapy, Department of Pharmaceutical and Pharmacological Sciences, KU Leuven (Catholic University of Leuven), B-3000 Leuven, Belgium; marianne.carlon@kuleuven.be; 4 Laboratory of Respiratory Diseases and Thoracic Surgery (BREATHE), Department CHROMETA, KU Leuven (Catholic University of Leuven), B-3000 Leuven, Belgium

**Keywords:** CFTR function, biomarker, cystic fibrosis, bioassay, anion channel

## Abstract

Cystic fibrosis, a multi-organ genetic disease, is characterized by abnormal function of the cystic fibrosis transmembrane conductance regulator (CFTR) protein, a chloride channel at the apical membrane of several epithelia. In recent years, therapeutic strategies have been developed to correct the CFTR defect. To evaluate CFTR function at baseline for diagnosis, or the efficacy of CFTR-restoring therapy, reliable tests are needed to measure CFTR function, in vitro, ex vivo and in vivo. In vitro techniques either directly or indirectly measure ion fluxes; direct measurement of ion fluxes and quenching of fluorescence in cell-based assays, change in transmembrane voltage or current in patch clamp or Ussing chamber, swelling of CFTR-containing organoids by secondary water influx upon CFTR activation. Several cell or tissue types can be used. Ex vivo and in vivo assays similarly evaluate current (intestinal current measurement) and membrane potential differences (nasal potential difference), on tissues from individual patients. In the sweat test, the most frequently used in vivo evaluation of CFTR function, chloride concentration or stimulated sweat rate can be directly measured. Here, we will describe the currently available bio-assays for quantitative evaluation of CFTR function, their indications, advantages and disadvantages, and correlation with clinical outcome measures.

## 1. Introduction

Cystic fibrosis (CF) is a multi-organ recessive genetic disorder caused by mutations in the *CFTR* (CF transmembrane conductance regulator) gene. These lead to the absence or dysfunction of the CFTR protein [1]. The CFTR protein is expressed at the apical epithelial cell membrane of most organs, where it functions as an ion channel. In the lungs of people with CF (PwCF), the decreased or absent epithelial chloride and bicarbonate secretion lead to airway surface liquid depletion and as a consequence, thick mucus, bronchial obstruction, and cycles of infection and inflammation. Ultimately this leads to chronic bacterial infection, causing bronchiectasis and respiratory failure, the most frequent cause of death [1]. In addition, disease or organ dysfunction occurs in the pancreas, gastrointestinal tract, liver, genital tract, sweat gland, and other organs.

In the last decade, the disease CF has entered a new era as several medicines that restore CFTR function have become available. This success is largely due to the enormous expansion of knowledge and detailed study of CFTR function using different models and assays. Correctors improve CFTR folding and trafficking so that more CFTR is available at the cell membrane, potentiators improve CFTR channel gating [2]. At present, the combination of the correctors tezacaftor and elexacaftor and the potentiator ivacaftor provides the best clinical improvement in PwCF who have at least one F508del mutation [1,3,4] and in subjects with a selected list of other mutations (in total 177 different mutations) [5]. More correctors and potentiators are under development. In addition, strategies to improve CFTR function in the group of subjects who do not derive benefit from current CFTR modulators are being explored: stop codon read-through compounds, correction of splicing by antisense oligonucleotides, the addition of mRNA, gene therapy, and eventually gene correction/editing by CRISPR/Cas [6,7,8].

CFTR function (net anion efflux) depends on the number of CFTR channels expressed at the apical membrane and on the function of each of these channels. The latter relates to the single-channel conductance (rate of ion transport across the apical membrane), which in turn depends on the electrochemical gradient (i.e., membrane potential and chloride concentration) across the membrane and on the open probability (P_o_) of the channels [9]. Depending on the type of *CFTR* mutation, one or more of the determinants of function are diminished and will be the main reason for the loss of function and thus reduction of anion secretion. In a nutshell, mutations grouped in classes I (stop codon mutations), II (defective processing), V (abnormal splicing), VI (instability at the cell membrane), VII (mutations not rescuable by compounds since no CFTR protein is produced, e.g., large deletions, insertions, frameshift mutations) will mainly decrease the number of CFTR channels, be it by different mechanisms. Mutations of class III decrease the open probability of the ion channel, and mutations of class IV reduce channel conductance [10]. However, this classification is an oversimplification as most mutations have characteristics of more than one mutation class [11].

Starting at the preclinical phase and continuing through all clinical phases of CFTR modulator development, there is a need for reliable tests to quantify CFTR function. Depending on the mutations of the specific target population, the aim will be to improve the number of CFTR channels at the cell membrane, the gating or conductance of the channel, or a combination of those. There is, however, no straightforward method to determine the exact number of CFTR channels at the membrane; only a relative quantification comparing samples to each other is possible [12,13]. We can, however, determine the total CFTR activity in a sample or study the channel function specifics.

In this paper, we thus review the main bio-assays available to study CFTR function. For each assay, we describe the main principle of the technique, cover its main indication, highlight its advantages and disadvantages, and, if applicable, future developments.

## 2. CFTR Bioassays

Not only different methods but also different samples can be used to study CFTR function either in vitro, ex vivo, or in vivo. Here we list just a few as the full range of possibilities is outside the scope of this review. As in vitro systems, animal cell lines that overexpress the human CFTR protein (heterologous systems) are available: baby hamster kidney cells (BHK), Chinese hamster ovarian cells (CHO), Fisher rat thyroid cells (FRT). In addition, epithelial or non-epithelial cell lines of human origin, overexpressing CFTR can be used, e.g., human embryonic kidney (HEK) cells, cervical cancer cells (HeLa), immortalized human CF bronchial epithelial cells (CFBE). Since the latter only minimally express CFTR endogenously, they have to be transfected with a plasmid or transduced with a viral vector encoding wild type or mutant *CFTR*. Next, primary epithelial cells and organoids derived from patients with CF and thus more representative of human samples can be studied: human nasal epithelial cells (HNE), human bronchial epithelial cells (HBE), colon and airways organoids. For a complete overview of the different CF cell models, we refer to the following review articles [7,14,15,16,17,18]. Ex vivo systems measure CFTR activity in tissue biopsies from human subjects or animal models. Finally, the human subject can be studied directly.

**Figure 1:**
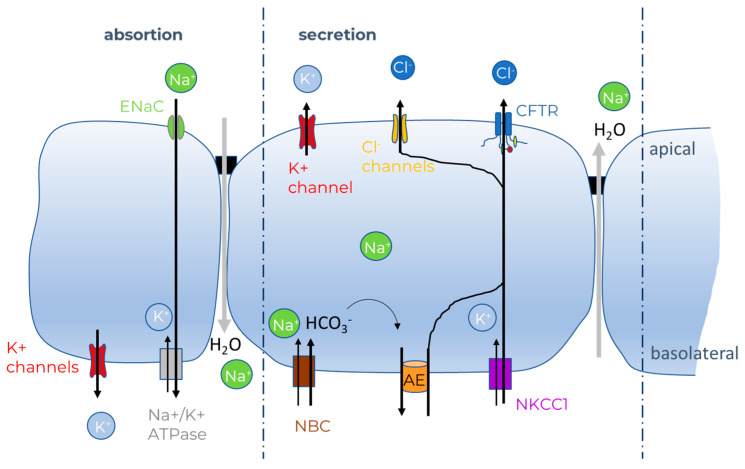
Diagram representation of ion transport in epithelial cells based on [19], for details, see text.

Generally speaking, salt and fluid transport are tissue-specific processes regulated through the involvement of ion channels, exchangers, cotransporters, and pumps. Ions and water move across epithelia either through the paracellular or transcellular pathways. Salt absorption is regulated mainly through the apically located epithelial Na^+^ channel (ENaC), which creates an electrochemical driving force for passive chloride (Cl^−^) transport from the luminal to the basolateral side (Figure 1). The salt concentration gradient in turn promotes passive water transport through osmosis. The basolateral Na^+^/K^+^-ATPase pump creates an inwardly directed sodium gradient to sustain absorption. Salt secretion, on the other hand, is mainly regulated by chloride channels and predominantly by CFTR, although calcium-activated chloride channels (CaCC) like anoctamin1 (ANO1 also known as TMEM16A) and solute carrier (SLC)26A9 are also involved. Chloride secretion begins with the intracellular accumulation of chloride mediated through the Na^+^ K^+^ 2Cl^−^ cotransporter (NKCC1) and the Na^+^/bicarbonate cotransporter (NBC), but also the Cl^−^/HCO_3_^−^ anion exchanger (AE) in some epithelial cells. Active chloride transport then creates the driving force for paracellular Na^+^ movement. Finally, the increased salt concentration on the luminal surface generates an osmotic driving force for water. Both for absorption and secretion, water moves either through the paracellular pathway or transcellular through aquaporins. Besides chloride, CFTR and ANO1 also conduct bicarbonate, which likewise regulates transepithelial fluid secretion. For a comprehensive overview of the role of CFTR in epithelial physiology, we refer to Saint-Criq V. and Gray M.A. [19] and Hanssens et al. [20].

### 2.1. In Vitro CFTR Bioassays

Several in vitro methods can be used to evaluate the function of CFTR as an anion channel: (1) evaluation of anion fluxes using iodide selective electrodes, radio-isotope tracers or fluorescent probes, (2) electrophysiological methods, based on the measurement of (i) the ionic current, or (ii) membrane potential across epithelia expressing apical CFTR [21].

#### 2.1.1. Ion Fluxes

- Iodide efflux

**Figure 2:**
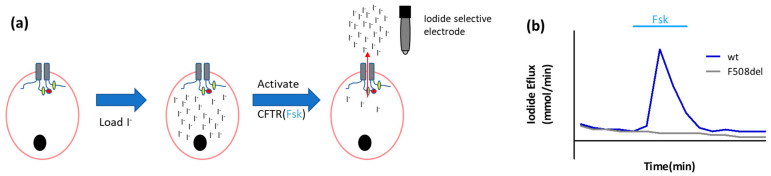
Iodide efflux assay with selective electrode. (**a**) Schematic representation of the iodide efflux set-up. After loading the cells with iodide and washing them, CFTR function is stimulated by forskolin (Fsk) for about 4 min. After the iodide loading and washing steps and with an interval of 1 min, the medium is refreshed and collected, starting a few minutes before CFTR stimulation with Fsk and ending some minutes after stopping CFTR stimulation. The efflux of iodide is measured in the samples using an iodide selective electrode. (**b**) The iodide efflux, as represented in the graph, quantitatively reflects the CFTR function in wild type compared to F508del mutant cells (illustrative tracing).

The CFTR channel is permeable to chloride and bicarbonate, but also to halides (iodide, bromide), that are not naturally present in the intra- or extracellular space. By using halides, CFTR permeability can be measured specifically, as other anion co-transporters are not halide-permeable. After loading cells with radioactive (^125^I^−^) or non-labeled iodide, CFTR is activated by forskolin, followed by quantifying the iodide efflux through the CFTR channel. This can be measured in different ways, one of them being the quantification of the fractional efflux over time of ^125^I^−^ transported into the medium with a gamma-radiation counter [22,23]. Alternatively, iodide selective electrodes can be used (Figure 2) [21,22,24]. In cells with normal CFTR function, an increase in iodide efflux will be measured after activation of the CFTR channel by forskolin (an adenylate cyclase agonist); in non-functional mutants like F508del, no iodide efflux will be detected over time.

The two described iodide efflux assays each have their pros and cons. For the radioactive version, the limitation is the requirement for authorizations to work with radioactive epitopes and the associated safety precautions. On the other hand, the sensitivity of this assay is high due to the absence of background signal as cells are not naturally radioactive. This is in contrast to fluorescent-based approaches where auto-fluorescence can interfere with the read-out. The iodide selective electrodes offer the advantage of being able to work without radioactive iodide. However, the electrodes can be unstable, so repetitive calibration is needed [21]. For both assays, a common advantage is their application in heterologous cell models overexpressing CFTR, which do not form a polarized epithelial monolayer, and hence do not allow typical transepithelial electrical measurements (see Section 2.1.2, subsection on transepithelial short circuit current (Isc) measurements). Technical aspects to be considered are cell densities, proper cell attachment, and proper washing of the iodide-loaded cells before the start of the assay to avoid residual signal.

This assay has been used in a wide variety of settings ranging from studying mechanisms of CFTR activation [23] or the effect of mutations in *CFTR* impairing channel function [25], to comparing *CFTR* mini-genes in the context of gene therapy [26].

- Halide-sensitive fluorescent probes

**Figure 3:**
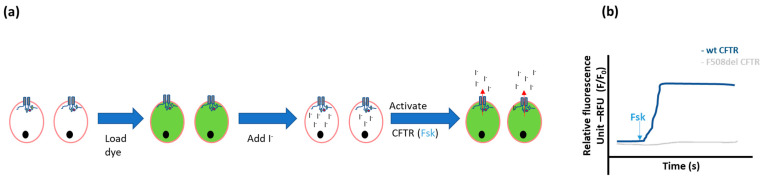
Schematic representation of the quenching assay. (**a**) First, cells are loaded with fluorescent probes, which induce fluorescence. Next, the cells are loaded with iodide, which induces quenching (i.e., decrease in fluorescence). After stimulation of CFTR by forskolin (Fsk), efflux of iodide through CFTR will increase the cell fluorescence again in the case of an active CFTR channel. (**b**) The fluorescence measured over time represents the CFTR function in wild type compared to F508del mutant cells (illustrative tracing).

Measuring efflux of halides through CFTR channels can also be performed using fluorescent molecules [27]. Several fluorescent probes are available: MQAE (N-(ethoxycarbonylmethyl)-6-methoxyquinolinium bromide), SPQ (6-methoxy-*N*-(3-sulfopropyl)quiolinium), and MEQ (6-methoxy-*N*-ethyl-1,2-dihydroquinoline) are chemical chloride or iodide indicators that quench (i.e., decrease in fluorescence) upon contact with halides. Similar to the measurement of iodide efflux, cells have to be loaded with the fluorescent probes and with iodide (Figure 3). When iodide is added, the fluorescence decreases but it increases again with the iodide efflux caused by stimulation of a functional CFTR channel by forskolin [28]. Iodide is preferred to chloride in these experiments since it is transported by CFTR but not by many non-CFTR chloride channels and the quenching is more pronounced with iodide compared to chloride, leading to a better signal/noise ratio [29].

This technique can be used to evaluate CFTR function in cultured epithelial cells, or ex vivo in freshly obtained primary human nasal or bronchial epithelial cells, for example to evaluate the effect of genes or gene-related therapy [28,30] or other CFTR modulating therapies [27,31,32,33]. This way, the individual response of patients to a CFTR modulator can either be predicted before in vivo application or monitored during therapy.

The advantages of this technique are that the measurements require very little material, can be performed on individual cells, and are even feasible on freshly isolated human cells [30]. Limitations of using fluorescent probes are limited repeatability and reproducibility due to leakage of dyes from the cells, photobleaching of the dye when prolonged periods of illumination are used, and the need for multiple loading and washing steps. The dynamic range of measurement is limited: very high or low fluxes cannot be detected reliably. Moreover, because of high variability, complex calibration procedures and controls are required.

- Halide-sensitive (HS) yellow fluorescent protein (YFP) quenching

To bypass the step of loading cells with fluorescent markers, cells can be genetically encoded with HS fluorescent proteins, such as YFP. Within the same cell, CFTR is either endogenously expressed or overexpressed to allow a functional assessment.

**Figure 4:**
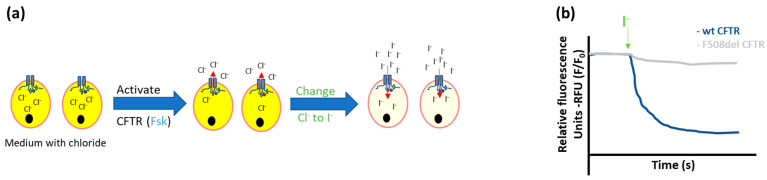
Schematic representation of the Halide Sensitive-YFP quenching assay. (**a**) If the YFP transfected cells are grown in a medium containing chloride, fluorescence will be present. Next, CFTR is activated by forskolin and the medium is changed from chloride- to iodide-rich. This induces an influx of iodide through activated CFTR and thus quenching of the YFP fluorescence. (**b**) The magnitude of the drop in fluorescence over time increases with increasing CFTR function, for example, in wild type compared to F508del mutant cells (illustrative tracings).

In this assay, a genetically modified YFP is used with a high quenching sensitivity to iodide, but very low sensitivity to chloride, which is generated by the introduction of key mutations (H148Q-I152L-F46L) in the YFP protein [34]. Cells co-expressing HS-YFP and a CFTR variant emit yellow fluorescence at baseline when analyzed under the microscope or by a specific plate reader. Next, depending on the CFTR variant studied, a pre-incubation step with a CFTR corrector for 24 h can be included. Twenty minutes before the measurement, forskolin and, if necessary, a potentiator are added, after which an iodide-rich buffer is added to the treated cells. HS-YFP quenching is monitored immediately after, typically over a short time period (Figure 4) [34].

This technique has been optimized for non-polarized cell lines, similar to the iodide efflux assay described above, although, it is not limited to these cell models only. In that light, it has been used for different applications such as high throughput screening of compounds to rescue CFTR function in various cell lines (FRT, HEK, CFBE) [13,34,35,36,37]. This technique has also been used to study the effect of (rare) mutations in CFTR channel gating [13] or to evaluate the effect of CFTR modulators on gating [38,39].

The HS-YFP quenching assay has several advantages compared to the two previous techniques: preliminary steps of loading the dyes and washing cells are not needed, the fluorescence remains stable over time, and therefore automation is feasible. In that light, results of a functional rescue in FRT cells expressing rare mutations have been the basis for expanding access to modulators in patients with these mutations [40,41]. A disadvantage, which is common to all tests using plasmid transfection or viral vector transduction in cell lines, is that both contain only the coding part of the *CFTR* gene (no introns are included). Thus, the results will not reflect the effect on CFTR function of mutations that impact splicing [25,42]. This can be overcome by transfection/transduction of the YFP protein in cells with endogenous expression of (mutated) CFTR.

- Fluorescent-based membrane potential assay

**Figure 5:**
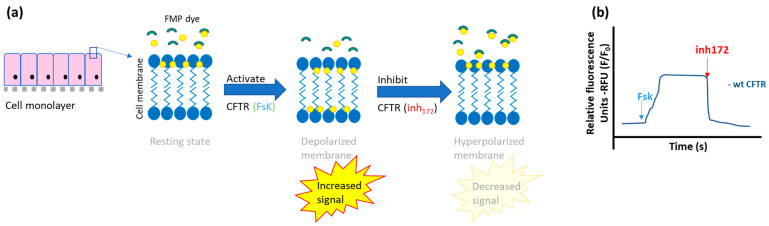
Schematic representation of the fluorescent-based membrane potential (FMP) assay. (**a**) The FMP dye combining a fluorescent dye and a quencher is added on top of the cell layer. With the apical membrane at rest state (baseline condition) there is some fluorescence from the dye. Activating CFTR by agonists depolarizes the membrane by increasing the negative charges at the outside due to the efflux of chloride through CFTR. This will, in turn, increase the fluorescent signal of the dye since it moves to the positive charges (inside), releasing the quenching. When CFTR is inhibited, the membrane becomes hyperpolarized, with an excess of positive charges outside decreasing the fluorescence signal due to the fact that the dye also moves outside, leading to increased quenching of the dye. Image based on supplier information (https://www.moleculardevices.com/products/assay-kits/ion-channel/flipr-membrane-potential#Technology; accessed on 6 January 2022) (**b**) Illustrative tracing of the fluorescence measured over time increases with increasing CFTR function upon addition of forskolin (fsk) in wild type mutant cells and decreases with the inhibition of CFTR by inh172.

An assay based on the change of membrane potential generated by the activation of CFTR was recently optimized to measure anion flux induced by CFTR [43]. In this assay, the membrane depolarization observed after activation of CFTR by agonists is measured by a fluorescent membrane potential (FMP) probe. This probe is loaded to the apical side and fluorescence increases when the membrane potential is altered (membrane depolarized) by activation of CFTR. Inversely adding a CFTR inhibitor will result in a reduction of fluorescence due to the hyperpolarization of the membrane (Figure 5).

Measuring the fluorescence alterations after the addition of CFTR agonists and/or inhibitors leads to quantification of CFTR activity and can be done in a kinetic way. Results obtained in primary nasal epithelial cells were shown to correlate with the results obtained in Ussing chamber measurements (see Section 2.1.2, subsection on the Ussing chamber).

This commonly called ‘fluorometric imaging plate reader’ (FLIPR) membrane potential assay has the advantage to measure efflux (depolarization) and influx (hyperpolarization of the membrane), in sequence, thus allowing identification of activators and inhibitors of CFTR much easier compared to the methods described before that mainly allows detection of CFTR activators. Another advantage is that the assay can be optimized to allow large-scale use in high-throughput techniques, both in non-polarized [44,45] and polarized epithelial cells [46], as well as in iPSC-derived lung progenitor cells and ciliated cholangiocytes from patients with CF [47,48]. The main disadvantage is that non-CFTR ion channels may also alter the membrane potential which can be overcome by using compounds with a CFTR specific action like the CFTRinh172 [44].

#### 2.1.2. Electrophysiological Methods

- Patch clamp technique

**Figure 6:**
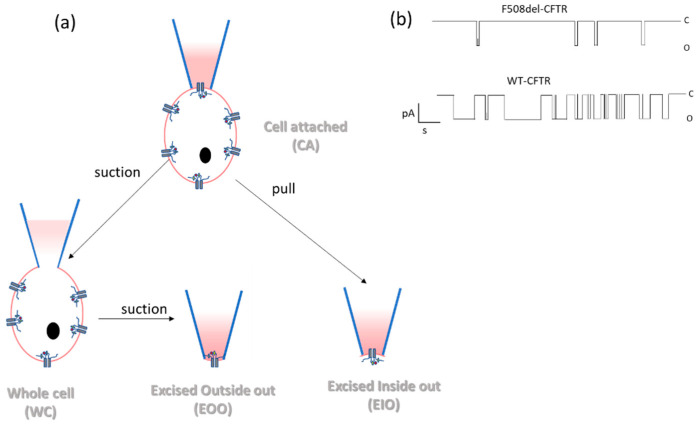
Patch clamp technique. (**a**) Diagram of the possible configurations (cell-attached, whole-cell, excised outside-out, and excised inside-out) for the patch-clamp assay, the inside-out configurations being the most used in CF research. (**b**) Simplified drawing of recordings of excised inside-out patches from heterologous cells (over)expressing wt-CFTR and F508del-CFTR (illustrative tracings of recordings based on [49]).

The patch clamp technique created such a multitude of possibilities that Neher and Sakmann, who developed it, obtained the Nobel prize for physiology in 1991. Indeed, ionic currents form the basis of many physiological processes (e.g. excitation of nerves, muscle and cardiac fibers, the function of ion channels…). Thus, an assay that measures ionic currents with high fidelity allows us to investigate the role of ion channels in (patho)physiological pathways. Patch clamping allows these currents to be measured accurately either by fixing the voltage (voltage clamping to measure ions flow through the channels, as mainly used in CFTR research) or by fixing the current (current clamping to determine the influence of the channels on the membrane voltage). To form an electrical circuit, a micropipette filled with electrolyte solution and a recording electrode is attached to the cell membrane and a second “reference” or “ground” electrode is placed in the bath surrounding the cell or tissue of interest [9]. The diameter of the micropipette and the patch configuration (cell-attached, whole-cell, outside-out or inside-out) determine whether the function of several or, in some cases, just a single ion channel will be evaluated (Figure 6).

The electrolyte solution in the micropipette and in the bath can be modified to allow various measurements: for whole cell recordings the micropipette solution is similar to the cytoplasm composition to create a more physiological background; for cell attached recordings the micropipette solutions must be similar to the bath solution only. The solutions can be further manipulated to avoid the contribution of sodium, potassium and calcium currents to the recording or to maximally activate CFTR channel function by, e.g., CFTR modulators. The whole cell configuration allows the “macroscopic” measurement of ionic currents resulting from all ion channels active at the plasma membrane, thus the best method to determine the overall function of CFTR in epithelial cells [50,51]. The cell-attached, outside-out and inside-out configurations allow recording a single-channel or just a few channels to provide a more refined “microscopic” evaluation of channel properties, like current amplitude and open and closed probabilities.

In CF research, the preferred method is the inside-out configuration whereby the patch is excised from the main body of the cell to study the behavior of individual CFTR channels by giving access to the intracellular side of the membrane. The wild type or mutant CFTR can thus be studied by patch clamp analysis to determine CFTR channel biophysical properties (e.g., conductivity and open probability) and how these are improved by CFTR modulators. These data then also contribute to understanding structure-function relations. Care must be taken to ensure that the characteristics of the channel measured are indeed those of the CFTR channel, such as ATP dependence, selectivity in anion conductance, linear voltage-current relationship [9].

The advantage of this assay is that the behavior of the CFTR chloride channel can be studied in great detail. Also, measurements can be done on sparse samples such as primary cells obtained from biopsies or brushings, as single cell measurements are performed. Furthermore, patch clamp can be done on both polarized as well as non-polarized cells. The limitations are that the technique is challenging, requires high expertise in physiology and is not easy to perform using cells with low expression of CFTR at the apical membrane such as primary epithelial cells. Although mainly CFTR overexpressing systems are used for this technique, it has recently been applied in primary nasal cells [52].

- Transepithelial short circuit current (Isc) measurements—Ussing Chamber

**Figure 7:**
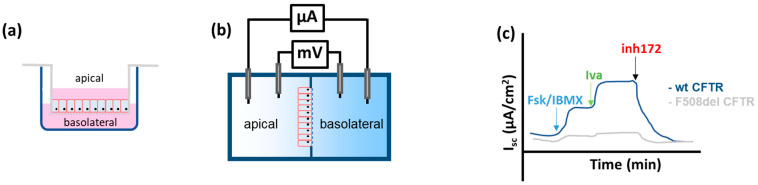
Isc measurements using the Ussing chamber. (**a**) The epithelial cells are grown on plastic porous supports (transwells), until they form a monolayer of confluent polarized cells. (**b**) Simplified drawing of the chambers separated by the monolayer of cells. One set of electrodes is placed near the cell monolayer to measure the voltage on both sides (potential difference-PD). This PD is then cancelled out (voltage clamped) by applying current (Isc) to the system using a second set of electrodes placed far from the cell monolayer. The Isc needed reflects the net ion transport across the cell monolayer (**c**) Illustrative example of a tracing for an epithelial cell monolayer expressing wildtype (wt)- or F508del-CFTR after activation by forskolin (Fsk) and 3-isobutyl-1-methylxanthine (IBMX), potentiation with ivacaftor (Iva) and inhibition by a CFTR inhibitor (inh172).

The Ussing chamber, developed in 1950 by the Danish physiologist Hans H. Ussing [53], provides a physiological system to measure the transport of ions, nutrients and compounds across an epithelium in native tissue or in a monolayer of polarized cells grown on permeable supports. The epithelial cells are grown on plastic porous supports (transwells), until they form a monolayer of confluent polarized cells and attain the transepithelial resistance appropriate for the cell type to allow transfer of cells to the Ussing chamber. The cell monolayers are then mounted in the Ussing chamber to measure the transepithelial short circuit current, i.e., the current across the epithelium when the transepithelial voltage is clamped to zero mV (Figure 7).

When studying CF epithelia, the main current of interest is the CFTR-dependent chloride current. However, under normal circumstances, ENaC contributes more or less to the Isc, depending on the tissue studied. Hence, to study the CFTR chloride current, the sodium transport at the apical side is inhibited by adding the ENaC blocker amiloride. The CFTR-dependent chloride current is low in resting conditions but can be activated when intracellular cAMP is increased by Fsk and IBMX, a phosphodiesterase inhibitor); the CFTR specificity of the elicited current can then be assessed by adding specific CFTR inhibitors like CFTRinh-172 [54]. In contrast to previously described functional assays, Ussing experiments require epithelial cell models which form an intact polarized monolayer with tight junctions, such as CFBE. However, also monolayers of epithelial cells with endogenous CFTR expression can be analyzed like primary airway epithelial cells from explant lungs (HBE), nasal brushings (HNE), colon organoids [46,55,56,57,58,59,60,61,62,63,64,65], or intestinal biopsies (see Section 2.2).

This Ussing technique is often considered the gold standard to further evaluate the efficacy of positive screens in high throughput assays since correction in Ussing chamber has proven to be a reliable predictor of in vivo benefit by the tested CFTR modulators [56,66,67]. The clinical phases of drug development of ivacaftor, lumacaftor, tezacaftor and elexacaftor for the most common mutation F508del was indeed guided by promising in vitro results in Ussing chamber during preclinical drug development [66,68]. Hence, the Ussing technique is now also used to determine the effect of compounds on other mutations. Cells expressing rare mutations can be pre-incubated with correctors or read-through compounds, or potentiators can be added acutely after CFTR activation by forskolin/IBMX [64].

The main advantages of this technique are that several cell types can be used, including primary epithelial cells with endogenous CFTR expression, not needing overexpression to quantify CFTR function and allowing usage of patient-derived samples. The limitations are the need for a high number of polarized epithelial cells, which are more difficult to reach when using nasal epithelial cells or primary bronchial cells.

- Forskolin-induced swelling (FIS) assay in organoids

**Figure 8:**
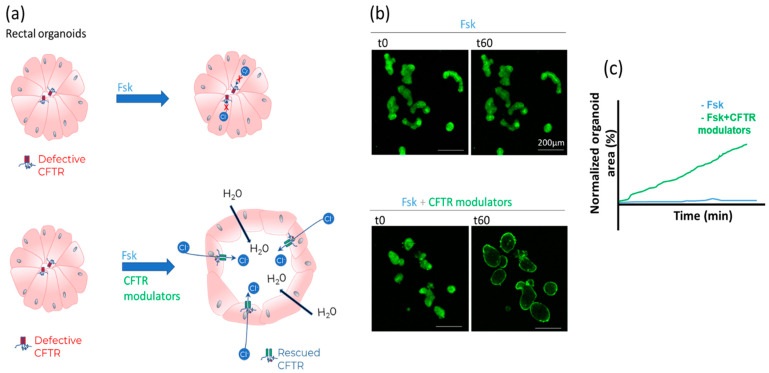
Forskolin-Induced Swelling (FIS) assay in rectal organoids from patients with CF. (**a**) Organoids are 3D structures of polarized cells with the apical membrane facing the lumen of the organoid. When the CFTR channel is defective, absent luminal secretion of chloride and concurrent water movement results in organoids with a slit-like or even absent lumen, even after addition of forskolin to activate CFTR. Rescue of CFTR function with modulators results in partial restoration of chloride transport and increase of the size of the lumen upon addition of forskolin. (**b**) Example of microscopic images of calcein stained organoids from an F508del homozygous patient at baseline (t0) and after (t60 min) activation of CFTR. In the upper panel only forskolin (Fsk) is added and no swelling is observed after 60 min. In the bottom panel, forskolin plus potentiator ivacaftor are added acutely and this after 24 h pre-incubation with correctors elexacaftor and tezacaftor. In this condition there is a considerable swelling of the organoids after 60 min, quantifying the rescue of F508del-CFTR function with this combination of compounds. (**c**) Illustrative tracing of organoid area increasing over time normalized to the area of the organoids at baseline (time zero).

The FIS assay in patient-derived organoids allows to quantify CFTR function and especially how it is corrected by CFTR modulators. Mostly intestinal organoids are used since CFTR is highly expressed at the apical (internal) membrane of the organoid, with very little expression of other ion channels. To obtain intestinal organoids, colon crypts are isolated from suction rectal biopsies, obtained via a painless procedure feasible at all ages. These crypts are rich in stem cells and by using specific media they can be grown into organoids, i.e., hollow 3D structures [69,70]. Upon activation of CFTR by forskolin, chloride is secreted into the lumen of the organoid, water and sodium follow passively, which results in swelling. Quantification of organoid swelling is thereby an indirect measurement of CFTR activity (Figure 8).

At baseline, organoids from subjects with CF are slit-like to ovoid in shape, in sharp contrast with the round organoids grown from healthy individuals. Rectal organoid morphology analysis (ROMA) could even be used as an ancillary diagnostic test for CF [71].

Organoids can be incubated with CFTR correctors, potentiators, amplifiers, read through drugs, gene therapies or any other drug thought to influence CFTR function before performing the FIS assay [26,72,73,74]. A good correlation has been demonstrated between mean improvement in lung function (FEV_1_, forced expired volume in one second) in clinical trials with CFTR modulators in patients with different genotypes and mean improvement in CFTR function in the FIS assay with these same drugs in organoids from patients with these same genotypes [73,75]. In patients with rare genotypes, a positive response in the FIS assay predicted clinical response to treatment [73,76].

The advantages of the FIS assay are that a patient-specific sample is used and that the rectum biopsies can be shipped to centers of expertise. Since the genetic background of the patient is represented in the organoid model, this is a practical approach towards personalized medicine, especially in patients with rare *CFTR* genotypes. Since it is unlikely that these patients will be included in clinical trials, and in vitro evaluation of potential treatments is the main way forward for them [77]. There are already several examples of the predictive value of organoid analysis for CFTR modulator use by individual patients or groups of patients [72,73,76]. The main limitations are that the technique is expensive (costly growth media, complex infrastructure, labor-intensive) and that a high level of expertise is required. In an endogenous CFTR expression system, there is always the influence of both *CFTR* alleles, an advantage as it reflects the patient’s overall CFTR function, but a disadvantage to attribute the defect or rescue to each individual mutation, especially if both lead to some CFTR expression (no class I or VII mutation) and/or when data from homozygous patients are not available. Also, organoids from subjects with high residual CFTR function are ‘pre-swollen’ which limits full assessment of CFTR functional rescue by modulators. For the same reason, FIS data in organoids cannot be expressed as a percent of wild-type function. However, since FIS responses in rectal organoids correlate well with Isc obtained from rectal organoid-derived epithelial monolayers [59], a comparison to WT is possible in the latter setting.

In recent years, work progressed in airway organoids derived from nasal polyps, nasal or bronchial brushing, cells recovered from bronchoalveolar lavage, explanted lungs, or differentiated from induced pluripotent stem cells [17,78]. Adaptations from the intestinal protocol are needed, mainly due to the slower growth and swelling rates of airway organoids [79]. The advantage of using respiratory cells is that the most diseased organ in CF is studied. One disadvantage is the slower growth rate which reduces the amount of material available for testing, thus lowering the number of drug combinations and replicates possible from the same patient sample. Airway organoids have a rather high expression of other ion channels, e.g., ENaC, which influence swelling, hence ENaC inhibitors like amiloride are needed in combination with forskolin and/or the assay must run over a longer period of time. Compared to intestinal organoids (the most advanced 3D model so far) the technique is less developed and more complex to establish [14]; however the correlation with other ion channels (like ENac and CaCC) is better represented in the 3D airway models [14]. Comparison between data obtained in intestinal and airway organoids is emerging [55].

### 2.2. Ex Vivo CFTR Bioassays

#### Intestinal Current Measurements (ICM)

**Figure 9:**
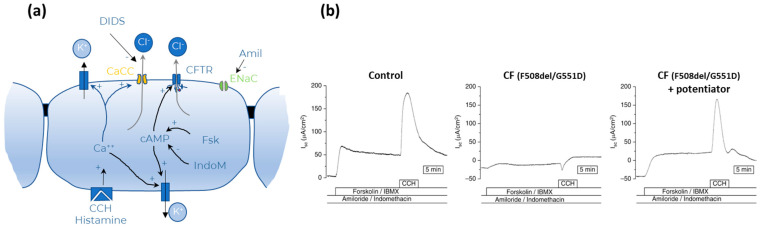
Intestinal current measurements (ICM) done on fresh rectal biopsies (**a**) Diagram of ion transport mechanisms with agonist and inhibitors and (**b**) illustrative recordings during ICM in a perfusing Ussing chamber in a person without (left tracing), with CF and a gating mutation without (middle tracing) and with the addition of the potentiator ivacaftor. See text for details. Tracings reprinted with permission from [82]. Copyright © 2022 American Thoracic Society. All rights reserved. The American Journal of Respiratory and Critical Care Medicine is an official journal of the American Thoracic Society. Readers are encouraged to read the entire article for the correct context at [82]. The authors, editors, and The American Thoracic Society are not responsible for errors or omissions in adaptations.

ICM is in essence an ex vivo application of the Ussing chamber technique (see Section 2.1.2, subsection on Ussing chamber). Currents or potential differences resulting from the ion fluxes are measured ex vivo in a small rectal biopsy mounted in a micro-Ussing chamber during the addition of pharmacological compounds to induce ion currents through CFTR. Normally, CFTR activity is stimulated by increasing cAMP synthesis by forskolin and decreasing cAMP degradation by IBMX, a phosphodiesterase inhibitor, after blocking ENaC with amiloride and inhibiting endogenous prostaglandin-mediated cAMP synthesis by indomethacin. This results in a sustained positive deflection in current if CFTR is functional. Addition of carbachol increases intracellular calcium concentration [80,81]. This elicits a potassium efflux through basolateral potassium channels, which in turn creates a favorable electrical gradient for apical chloride efflux via CFTR. In subjects with preserved CFTR function, a large “positive” deflection in short circuit current induced by chloride efflux masks the smaller “negative” deflection in current caused by calcium-dependent potassium efflux, which can only be seen if CFTR function is absent (Figure 9). Other protocols add DIDS to inhibit calcium-activated alternative chloride channels (CaCC) and/or histamine to increase intracellular calcium concentration.

Standard operating procedures (SOPs) were established, with still two main experimental setups possible, reperfusion and continuous perfusion chambers. In the former, the solutions bathing the biopsy are recirculated and compounds added remain in the system during the entire experiment. In the latter, the solutions are perfused in an open system, thus requiring larger amounts but allowing removal of compounds during the procedure. To reduce variability, results from three to four biopsies were averaged, typically the sum of the response to forskolin/IBMX and to carbachol/histamine. Reference values have been established in CF and non-CF subjects [83].

The main use of ICM has been as a diagnostic aid in equivocal cases of CF [84]. More recently, ICM has also been used to assess improvement in CFTR function by modulators in an individual ex vivo. Ivacaftor was shown to partially correct CFTR function in rectal biopsies of patients with the G551D mutation [82]. In a number of patients with rare and incompletely characterized mutations, ICM could be used to evaluate the change in CFTR function by CFTR-directed therapies [85].

An advantage is that the equipment is rather inexpensive. In addition, using freshly taken biopsies allows assessing drug effects in “near-body conditions”. After the ICM, the biopsies can be put in culture to grow rectal organoids (see section on FIS assay) that can be stored and used for further evaluation of CFTR-directed therapies. The main limitation of using ICM to assess the benefit from modulators is the fast decline of CFTR function in the biopsies, losing half of it in 24 h [86]. This implies that only a limited number of treatments can be tested on each biopsy. ICM is therefore also less suited to test correctors with a typically slower mode of action requiring drug exposition for many hours, unless the patient was already treated with the corrector in vivo. Mostly, biopsy and analysis need to be performed at the same site, even if some experience in remote processing of biopsies has been gained in a multicenter trial [86]. The availability of this methodologically complex assay is indeed very limited and restricted to a small network of specialized labs.

### 2.3. In Vivo CFTR Bioassays

#### 2.3.1. Sweat Chloride Concentration (SCC)

**Figure 10:**
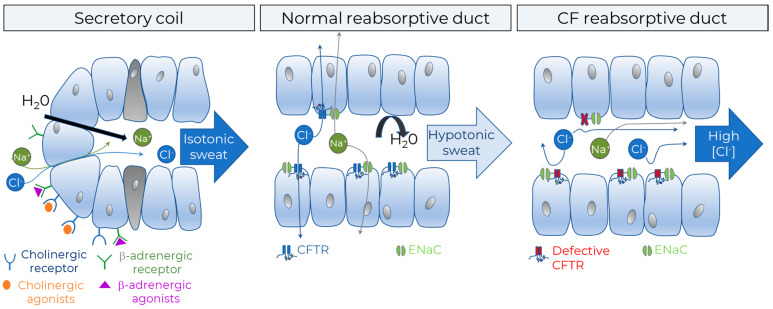
Sweat test, with a schematic representation of the secretory coil (**left panel**) and the absorptive duct from the sweat gland (SG) of a person without CF (**middle panel**) and a person with CF (**right panel**). After stimulating sweat secretion by pilocarpine, isotonic sweat is produced in the sweat coil. In the non-CF SGs, chloride is reabsorbed through CFTR in the sweat duct, sodium follows through ENaC, but not water, resulting in hypotonic sweat with a low chloride concentration. In SGs from people with CF, CFTR is defective and sweat chloride is not/poorly reabsorbed, resulting in sweat with a high chloride concentration.

In people without CF, isotonic sweat is produced in the coil of eccrine sweat glands and chloride is subsequently reabsorbed through CFTR in the sweat duct (Figure 10). In PwCF, chloride is not reabsorbed, leading to a higher sweat chloride concentration (SCC). SCC thus reflects CFTR function. Throughout different *CFTR* genotypes, there is a logarithmic correlation between the mean SCC reported in registries and CFTR function measured in FRT cells [87]. Sweat induction by pilocarpine iontophoresis, sweat collection, and measurement of SCC have been fully standardized [88].

The main use of the sweat test is as the first-line diagnostic tool [84,89]. A SCC below 30 mmol/L makes CF unlikely, while a SCC of 60 mmol/L or more is diagnostic for CF. SCC has, however, been extensively used as a biomarker of CFTR function in clinical trials with CFTR modulators and this strategy has proven very successful [90]. The change in SCC already occurs within days to weeks after starting modulator therapy [4,91], and is maintained over the ensuing months [92,93]. Early reports of the lack of a correlation between individual changes in SCC and changes in FEV_1_ cast doubt on the validity of SCC as a biomarker in modulator trials [94]. Later, a correlation between the mean changes in SCC and in lung function parameter FEV_1_ was demonstrated using pooled data from different clinical trials. However, the correlation was not significant within each treatment/genotype group [95]. This lack of close correlation with the validated surrogate outcome measure FEV_1_ impairs qualification of SCC as a surrogate clinical endpoint [96].

The main advantages of the sweat test are that it is painless, cheap and easy to perform the test, requiring minimal equipment, being widely available and feasible at all ages. It has proven rapid responsiveness to intervention and concordant with clinical benefit in the context of clinical trials [95]. Given the feasibility, availability and responsiveness, SCC has and will continue to be used in modulator trials of all types, including proof-of-concept trials and even personalized n-of-1 trials [97,98]. The disadvantage is that the measurement of SCC does not reflect the respiratory system and is only of use to evaluate modulator therapies administered systemically but not those given via inhalation.

#### 2.3.2. β-Adrenergic Sweat Test

**Figure 11:**
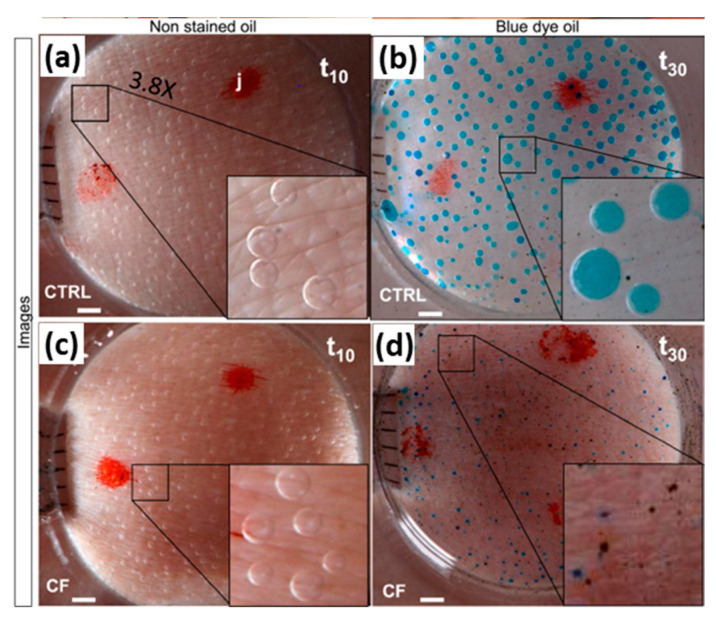
Image-based β-adrenergic sweat test. The cholinergic sweat pathway is stimulated by iontophoresis of pilocarpine; sweat secretion is captured by serial images over 10 min (p-phase). The β-adrenergic sweat pathway is then stimulated by a second iontophoresis/injection of a solution applied to the same skin site and containing atropine, aminophylline and isoproterenol plus ascorbic acid; images are then recorded over 30 min (β-phase). A Plexiglas circular well (2.27 cm^2^) is placed over the stimulated skin area (anode) and filled with a thin layer of unstained or erioglaucine blue-stained water-saturated mineral oil. Images are shown of sweat bubbles formed under the oil layer recorded at 10 min of the p-phase in a control (**a**) and in a patient with CF (**b**) and at 30 min of the β-phase in a control (**c**) and in a patient with CF (**d**). In panel (**a**), the letter “j” is one of the marks used to align successive images. Calibration bar: 1 mm. Inserts in panels (**a**–**d**) magnified 3.8×. figure reprinted with permission from Journal of Cystic Fibrosis. Copyright © 2022. All rights reserved. Readers are encouraged to read the entire article for the correct context at [105]. The authors, editors, and Journal of Cystic Fibrosis are not responsible for errors or omissions in adaptations.

Human sweat can be produced via cholinergic (control of thermoregulation) as well as β-adrenergic (fight and flight response) stimulation (Figure 10 and Figure 11). Since Sato and Sato [99] discovered the deficiency of the adrenergic sweat pathway in people with CF, assays have been developed to capture this difference between people with and without CF. In these tests, the non-affected cholinergic pathway is activated by intradermal injection of methacholine, resulting in an increase in M (methacholine)-sweat with no difference expected between CF and non-CF. Subsequently, the β-adrenergic sweat production is stimulated by intradermal injection of an adrenergic cocktail and a clear difference is observed: C-sweat, i.e., CFTR-dependent sweat is (near) absent in CF. Two main techniques are available. The evaporimeter calculates transepithelial water loss after adrenergic stimulation as a proxy of sweat rate via two probes on the forearm that measure temperature and humidity [100]. The even more sensitive image-based technique quantifies the sweat bubble volumes at the skin surface during M and C induced sweating. As main read out the ratio of C/M sweat is used to compensate for differences in sweat production between subjects [101,102,103]

Measurement of β-adrenergic sweat rate can be used as an ancillary test to diagnose CF and can distinguish between CF carriers and healthy controls. In preliminary experiments, the bubble test has also been used to demonstrate the appearance of C-sweat in a few subjects with ivacaftor responsive mutations [104].

Whereas the SCC measurement has a logarithmic correlation with % CFTR function and thus becomes insensitive at higher levels of function, the C/M ratio in the β-adrenergic sweat rate test has a near linear correlation over the entire dynamic range of CFTR function. This is the main advantage of the β-adrenergic sweat test. However, the limitations are that it requires a high level of expertise, complex equipment, repeated intradermal drug injections and long periods of inactivity of the arm, making the test less acceptable in children. The correlation between the β-adrenergic sweat test and other CFTR biomarkers has not been studied so far. Further validation is needed, in particular in pediatric cohorts. The technique is still being further refined and simplified. As in the classical sweat test, the M-sweat production can be stimulated by iontophoresis with pilocarpine and atropine [104], but is more complex for the adrenergic cocktail [105]. The set-up has been adapted to allow movement [106,107].

#### 2.3.3. Nasal Potential Difference (NPD)

NPD is an indirect assessment of the ion transport in the nasal airway epithelia: the change in voltage reflecting the change in current. The technique was first described by Knowles et al. [108], and was subsequently optimized and standardized [109,110,111,112,113,114].

**Figure 12:**
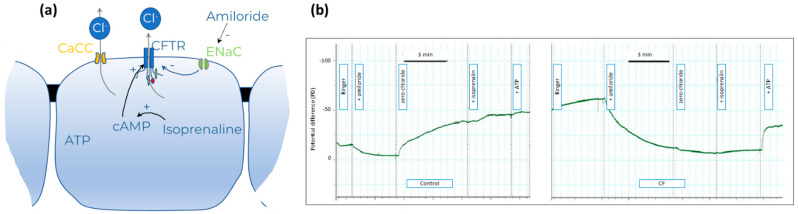
Nasal Potential Difference measurement. (**a**) Diagram of ion transport mechanisms during NPD measurements. (**b**) Representative tracing in a healthy control (**left panel**) and a person with cystic fibrosis (**right panel**). Reprinted with permission by the author [117]. See text for details.

Using calomel electrodes, the potential difference is measured between an agar-filled catheter in the nose and one in the isoelectric subcutaneous space used as a zero reference. During the measurements the nose is being perfused through the catheter sequentially by Ringer’s solution to assess the baseline NPD, Ringer’s solution with amiloride to measure changes in NPD after blocking ENaC and a zero-chloride solution with amiloride followed by a zero-chloride solution with amiloride and isoproterenol to stimulate CFTR function, the sum of the latter changes in NPD called “total chloride response” (Figure 12). ATP is then added to the solution to stimulate alternative chloride channels.

Compared to healthy mucosa, the CF nasal epithelium is characterized by decreased or absent CFTR function and secondary ENaC hyperfunction. This results in increased sodium absorption over the respiratory epithelia and thus hyperpolarized (more negative) baseline NPD, excessive depolarization in response to sodium channel inhibitors, such as amiloride, and little to no active chloride (Cl^−^) secretion in response to chloride-free solution and isoproterenol (Figure 12). Composite scores combining the ENaC and CFTR readout improve the sensitivity and specificity of NPD to discriminate between CF and healthy [115,116].

NPD is mainly used as an ancillary test to support or exclude the diagnosis of CF in subjects with intermediate sweat chloride values or when *CFTR* mutations of unknown significance have been identified [115,116]. Early on, NPD was used as an outcome parameter in clinical trials with systemic CFTR modulators. However, in the phase 2 trial with ivacaftor in subjects with G551D, the mean change in sweat chloride concentration (−60 mmol) and the mean improvement in FEV_1_ (+8.7%) were much more impressive than the mean change in the baseline NPD (−3.5 mV) [118]. In a prospective open-label observational study, a mean −1.5 mV change in total chloride response was measured in patients homozygous for F508del starting treatment with lumacaftor-ivacaftor [119]. Given the limited responsiveness of NPD (compared to other biomarkers) to intervention with potent modulators, in combination with the complexity and duration of the measurement, NPD has not been taken forward in modulator trials unless drugs were administered by inhalation [120,121]. So far, there is not yet an example of a proof-of-concept trial where the biomarker NPD successfully predicted clinical benefit in the further development of the inhaled modulator.

The advantage of NPD as a biomarker is that it assesses epithelial chloride and sodium transport in vivo, both being disturbed in CF. NPD has proven valuable as an ancillary test in the diagnostic pathway of CF. The limitations are the absence of commercial kits, the high intra-subject variability [111,122,123] the limited availability and the poor applicability in young children. Taken together, although NPD retains a place in the diagnostic path of CF, it is not a biomarker of choice in the development of systemic CFTR modulators.

See Table 1 for a summary of key references for all the protocols of the assays described in this review.

## 3. Conclusions

The CFTR protein is an anion channel expressed at the apical cell membrane of many tissues and its dysfunction is the cause of the wide range of disease manifestations seen in PwCF. Many assays have been developed to study CFTR function and how it can be improved by compounds in an effort to cure the CF. Each of these assays has a specific use, either to establish the diagnosis of CF, in the quest for CFTR modulators that improve the underlying disease process, or it can even serve both purposes. Generally speaking, the in vitro assays using ion fluxes are most adapted for high throughput screening of large libraries of compounds, whereas those that employ electrophysiological methods are more suitable for a detailed understanding of the function of wild type or mutant protein and its correction by modulators preselected by high throughput screening. The correction of F508del-CFTR function by CFTR modulators obtained in Ussing chamber experiments on native tissue has proven highly predictive of an in vivo benefit in a large group of PwCF carrying this most common mutation F508del. Primary epithelial cells (e.g., intestinal, nasal) grown as organoids or as a polarized monolayer on permeable support are, however, the most practical way forward for personalized medicine in the residual group of PwCF who have rare or ultra-rare *CFTR* mutations because they allow easy access to patient material that can be used over a longer period of time. Ex vivo and in vivo tests are available to aid in the diagnosis of CF: here, the sweat test remains the first-line diagnostic test, whereas ICM and NPD are reserved to confirm or exclude the diagnosis of CF in patients with suggestive symptoms but an inconclusive sweat test result. To this purpose also, beta-adrenergic sweat test or even in vitro organoid morphology (ROMA) can contribute. The sweat test is also widely used as a biomarker in the clinical phase of drug development, at least for compounds administered systemically: the global availability, strict standardization, fast response to the intervention being its major assets. Hence, depending on the question to be answered and the material available. The most appropriate CFTR function test can be chosen.

## Figures and Tables

**Table 1:** Key references of the protocols of each bioassay described in this review.

	Assay	Key References
In vitro bio-assays	Iodide efflux	Norez et al., 2004 [22], Long, K.J.; Walsh, K.B. 1997 [24]
	Halide-sensitive Fluorescent probes	Munkonge, F. et al., 2004 [30]
	Halide-sensitive yellow fluorescent protein (YFP)	Galietta, L.V.J. et al., 2001 [34]
	Fluorescent-base membrane potential probe	Maitra, R. et al., 2013 [43]
	Patch clamp CFTR functional analysis	Cai, Z. et al., 2011 [9] Sheppard, D.N. et al., 2004 [50]
	Transepithelial short circuit current (Isc) measurements	Li, H. et al., 2004 [54]
	FIS assay on rectal organoids	Dekkers, J.F. et al., 2013 [69] Vonk, A.M. et al., 2020 [70]
Ex vivo bio-assays	Intestinal current measurements (ICM)	Mall, M. et al., 2004 [81] De Jonge, H.R. et al., 2004 [80] Derichs, N. et al., 2010 [83]
In vivo bio-assays	Sweat chloride concentration	LeGrys, V.A. et al., 2007 [88]
	β-adrenergic sweat assays	Quinton, P. et al., 2012 [100] Wine, J.J. et al., 2013 [101]
	Nasal potential difference (NPD)	Knowles, M.R. et al., 1995 [108] Solomon, G.M. et al., 2018 [114]

## Data Availability

Not applicable.
